# 

**Published:** 1855-01-01

**Authors:** 


### THE JOURNAL

OF

### PSYCHOLOGICAL MEDICINE

AND

### MENTAL PATHOLOGY.

S.G.O.L. INDEXED

EDITED BY

FORBES WINSLOW, M.D., D.C.L. Oxon.

VOL. VIII.

Surgeon Genl's Office LIBRARY. 22052 Washington, D.C.

LONDON:

JOHN CHURCHILL, NEW BURLINGTON STREET.

MDCCCLV.

W
1
JO857N

LONDON

SAVILL AND EDWARDS, PRINTERS, CHANDOS-STREET,

COVENT GARDEN.

The parcel of books sent by Dr. Girolami, from Pesaro, we were obliged to refuse, in consequence of several pounds postage being demanded!

The excellent and well-written work on "Unsoundness of Mind in relation to Criminal Acts," by Dr. J. C. Bucknill, M.D., and the valuable Essay by Dr. J. W. Williams on "Unsoundness of Mind in its Medical and Legal considerations," published at length in the "Dublin Quarterly Journal of Medical Science," will be reviewed conjointly in our next number.

